# Role of PINCH and Its Partner Tumor Suppressor Rsu-1 in Regulating Liver Size and Tumorigenesis

**DOI:** 10.1371/journal.pone.0074625

**Published:** 2013-09-18

**Authors:** Shashikiran Donthamsetty, Vishakha S. Bhave, Wendy M. Mars, William C. Bowen, Anne Orr, Meagan M. Haynes, Chuanyue Wu, George K. Michalopoulos

**Affiliations:** 1 University of Pittsburgh School of Medicine, Department of Pathology, Pittsburgh, Pennsylvania, United States of America; 2 Philadelphia College of Osteopathic Medicine, School of Pharmacy, Department of Pharmaceutical Sciences, Suwannee, Georgia, United States of America; National Cancer Institute, United States of America

## Abstract

Particularly interesting new cysteine-histidine-rich protein (PINCH) protein is part of the ternary complex known as the IPP (integrin linked kinase (ILK)-**P**INCH-**P**arvin-α) complex. PINCH itself binds to ILK and to another protein known as Rsu-1 (Ras suppressor 1). We generated PINCH 1 and PINCH 2 Double knockout mice (referred as PINCH DKO mice). PINCH2 elimination was systemic whereas PINCH1 elimination was targeted to hepatocytes. The genetically modified mice were born normal. The mice were sacrificed at different ages after birth. Soon after birth, they developed abnormal hepatic histology characterized by disorderly hepatic plates, increased proliferation of hepatocytes and biliary cells and increased deposition of extracellular matrix. After a sustained and prolonged proliferation of all epithelial components, proliferation subsided and final liver weight by the end of 30 weeks in livers with PINCH DKO deficient hepatocytes was 40% larger than the control mice. The livers of the PINCH DKO mice were also very stiff due to increased ECM deposition throughout the liver, with no observed nodularity. Mice developed liver cancer by one year. These mice regenerated normally when subjected to 70% partial hepatectomy and did not show any termination defect. Ras suppressor 1 (Rsu-1) protein, the binding partner of PINCH is frequently deleted in human liver cancers. Rsu-1 expression is dramatically decreased in PINCH DKO mouse livers. Increased expression of Rsu-1 suppressed cell proliferation and migration in HCC cell lines. These changes were brought about not by affecting activation of Ras (as its name suggests) but by suppression of Ras downstream signaling via RhoGTPase proteins. In conclusion, our studies suggest that removal of PINCH results in enlargement of liver and tumorigenesis. Decreased levels of Rsu-1, a partner for PINCH and a protein often deleted in human liver cancer, may play an important role in the development of the observed phenotype.

## Introduction

There has been a long standing question in liver biology as to what regulates the size of the liver. It has been observed in experimental and clinical studies that a liver from a small donor, transplanted into a much larger recipient, rapidly increases in size and achieves a size comparable to that of a normal liver for that particular recipient in a period of 2 weeks. These phenomena have been extensively studied in dogs in which small-for-size livers were transplanted into larger recipient [1,2]. Similarly, when a baboon liver was transplanted to a human, the transplanted intact liver of the baboon rapidly grew in size until it reached the size of human liver, indicating that the size of liver can be also controlled by a mechanism outside the transplanted liver that is intrinsic to the host [3].

Recent studies in our lab have shown the important role of extracellular matrix (ECM) signaling via the Integrin Linked Kinase-PINCH-Parvin (IPP) complex in regulating the size of the liver. We have previously generated hepatocyte-specific integrin linked kinase (ILK) KO mice. The impact of this deletion to the liver has given us many exciting results related to the role of ECM in regulating liver size and termination of liver regeneration after partial hepatectomy (Phx) [4,5,6]. Liver histology in the ILK KO mice at 6 weeks and thereafter showed increased numbers of hepatocytes in mitosis and apoptosis. By the end of 30 weeks, livers of ILK KO mice are almost 30% larger than the control mice. These livers also do not properly terminate regeneration, and liver gains about 30% of weight when regeneration is complete [6]. While the studies with ILK ascertain the role of one of the components of the IPP complex in regulating liver size and termination of liver regeneration, the role of the other components in this process is not clear. The objective of the present study whether other components of IPP complex besides ILK also have a role in regulating the size of the liver. In this study, we concentrated on Particularly Interesting New Cysteine-Histidine-rich protein (PINCH) in regulating liver size. PINCH consists of five LIM domains each with unique sequences, and lacks a catalytic domain [7]. Following the discovery of PINCH (PINCH-1), a related protein PINCH-2, has been characterized. The two proteins share 82% amino acid sequence homology but are encoded by separate genes. Although PINCH-1 and -2 are co-expressed, they appear to be functionally distinct. Although PINCH1 has no catalytic abilities, the IPP complex serves as a link between integrins and components of growth factor receptor kinase and GTPase signaling pathways [7]. Accordingly, PINCH1-mediated signaling induces cell migration, spreading, and survival [7]. PINCH-2 on the other hand is potentially involved in mediating the PINCH-1/integrin linked kinase (ILK) interaction [7,8]. PINCH1 deficient mice are embryonically lethal [9] while mice deficient in PINCH2 are viable, fertile and exhibit no overt phenotype because PINCH1 substitutes for PINCH2 [10]. For this purpose we generated mice with targeted deletion of PINCH 1 to hepatocytes while PINCH2 was systemically eliminated through the germ line [6] (referred to as PINCH DKO mice). Our studies here show that these mice are born normal and the final liver weight by the end of 30 weeks in livers with PINCH1 and 2 deficient hepatocytes is 40% larger than the control mice (the size is even larger than the ILK hepatocyte-KO mice). By end of first year, some of the PINCH DKO mice develop spontaneous tumors. One mechanism through which loss of PINCH 1 and 2 may contribute to such a phenotype may involve the protein called Ras suppressor or Rsu-1. Studies have shown that Rsu-1 binds the LIM5 domain of PINCH-1 [11,12]. It was also shown that association of full-length Rsu-1 with IPP complex through PINCH1 correlates with reduced Ras transformation [11,12]. Rsu-IPP complex formation appears to promote adhesion, thereby decreasing migration, pointing to the importance of PINCH in facilitating the protein-protein interactions, which ultimately mediate cell behavior and fate. We have recently found that PINCH binding partner protein Rsu-1 is frequently deleted in human liver cancers [13]. In the present study we found that PINCH DKO mice have reduced levels of Rsu-1. Further, overexpression of Rsu-1 in hepatoma cell lines leads to its association with PINCH1 followed by reduced cell proliferation and migration. This study shows that Rsu-1, whose expression is dramatically downregulated in the PINCH DKO mice, is as potentially a key molecule by which PINCH (and IPP complex) could exert its growth suppressing effects on hepatocytes. Thus, our present studies show that PINCH and Rsu-1 are a part of the growth termination signaling of IPP which mediates signaling of extracellular matrix and integrins and which is activated in conjunction with termination of liver regeneration.

## Materials and Methods

### Ethics Statement

Animals (mice) were housed and treated according to institutional guidelines of the University of Pittsburgh IACUC committee (Protocol 1105844). This follows guidelines established by NIH for ethical use of animals in biomedical research. The IACUC committee and the aforementioned protocol specifically approved this study.

### Generation of PINCH1 and 2 double KO (PINCH DKO) mice

PINCH1 homozygous floxed animals harboring one copy of the afp/alb cre transgene were crossed with PINCH1^loxP/loxP^ homozygous animals already in a PINCH2 systemic KO background (imported from UCSD [14]). The resultant animals (PINCH1 homozygous flox and PINCH2 heterozygous, as well as either + or - for the cre) were crossed to obtain animals that were PINCH1 homozygous flox, PINCH 2 KO, and either + or - for cre. The PINCH1 ^loxP/loxP^ and PINCH2 KO with Cre were considered DKO mice. Cre negative mice were used as Control mice (PINCH2^-/-^ PINCH1 ^loxP/loxP^ Cre^-^). All animals were housed in the animal facility of the University of Pittsburgh in accordance with the guidelines of the Institutional Animal Use and Care Committee of the University of Pittsburgh.

### Antibodies and Commercial kits

The following antibodies were used in this paper: ILK (abcam ab52480), Rsu-1 (abcam, ab69843), PINCH (BD bioscience, 612710), alpha Parvin (Santa Cruz, 50693), SMA (Dako, M0851), Ki67 (Thermofisher, RM9106), HNF1β (Sigma, HPA002083), F4/80 (Abcam, ab6640) and turbo-GFP (Origene, TA150041). RhoA GST pull down assay was performed using a kit from Pierce (16116Y). Rac/cdc42 pull down assays were performed using kit from Millipore (17441). ROCK kinase assay was performed using a kit from Cell Biolabs (STA 415). GFP tagged ORF clone of Homo sapiens Rsu-1 (#RG203334, Origene) was purchased from Origene.

### Isolation of Hepatocytes

Hepatocytes from the rats, PINCH DKO mice as well as their respective controls were isolated by an adaptation of the calcium two-step collagenase perfusion technique. After the liver perfusion, hepatocytes were separated from the nonparenchymal cells of the liver by several centrifugation steps. Briefly, the cell pellet obtained from the liver perfusion was centrifuged at 1,000 *g* for 5 minutes. The pellet was washed subsequently with Hank’s buffered salt solution and centrifuged at 1,000 *g* for 5 minutes, the pellet was kept as the fraction that corresponds to hepatocytes.

Protein isolation and Western Blotting: Total protein was isolated from the mouse hepatocytes or whole livers from the PINCH DKO and control mice using 1% sodium dodecyl sulfate (SDS) in RIPA buffer (20 mM Tris/Cl pH 7.5, 150 mM NaCl, 0.5% NP-40, 1% TX-100, 0.25% Sodium Deoxycholate (DOC), 0.6-2 µg/ml aprotinin, 10µM Leupeptin, 1µM Pepstatin). Protein concentrations of all lysates were determined using the bicinchoninic acid protein assay reagents (BCA method) (Pierce Chemical Co., Rockford, IL). Nuclear proteins were prepared using the NE-PER nuclear and cytoplasmic protein isolation kit (Pierce, Rockford IL) according to the manufacturer’s protocol. Total cell lysates made in Ripa buffer (50 µg) were separated by sodium dodecyl sulfate polyacrylamide gel electrophoresis in 4% to 12% NuPage Bis-Tris gels with MOPS buffer (Invitrogen, Carlsbad, CA), then transferred to Immobilon-P membranes (Millipore, Bedford, MA) in NuPAGE transfer buffer containing 20% methanol. Membranes were stained with Ponceau S to verify loading and transfer efficiency. Membranes were probed with primary and secondary antibodies in Tris-buffered saline Tween 20 containing 5% nonfat milk, then processed with SuperSignal West Pico chemiluminescence substrate (Pierce, Rockford, IL) and exposed to a X-ray film (Lab Product, Sales, Rochester, NY).

Imunohistochemistry: Paraffin-embedded liver sections (4 µm thick) were used for immunohistochemical staining. Antigen retrieval was achieved by heating the slides in the microwave at high power in citrate buffer for 10 minutes. The tissue sections were blocked in blue blocker for 20 minutes followed by incubation with pertinent primary antibody overnight at 4°C. The primary antibody was then linked to biotinylated secondary antibody followed by routine avidin-biotin complex method. Diaminobenzidine was used as the chromogen, which resulted in a brown reaction product.

GST pull down assays: GTP bound (active) RhoGTPase were measured in the whole cell lysates by GST pull down Assay. RhoA GST pull down assay was performed using a kit from Pierce (16116Y). Rac/cdc42 pull down assays were performed using kit from Millipore (17441). ROCK activity was measured by in vitro kinase assay by measuring the ability of ROCK to inactivate myosin phosphatase through the specific phosphorylation of myosin phosphatase target subunit 1 (MYPT1) at Thr696 (Cell Biolabs, STA 415).

## Results

### Components of the IPP complex are upregulated at the end of liver regeneration

**Figure 1:**
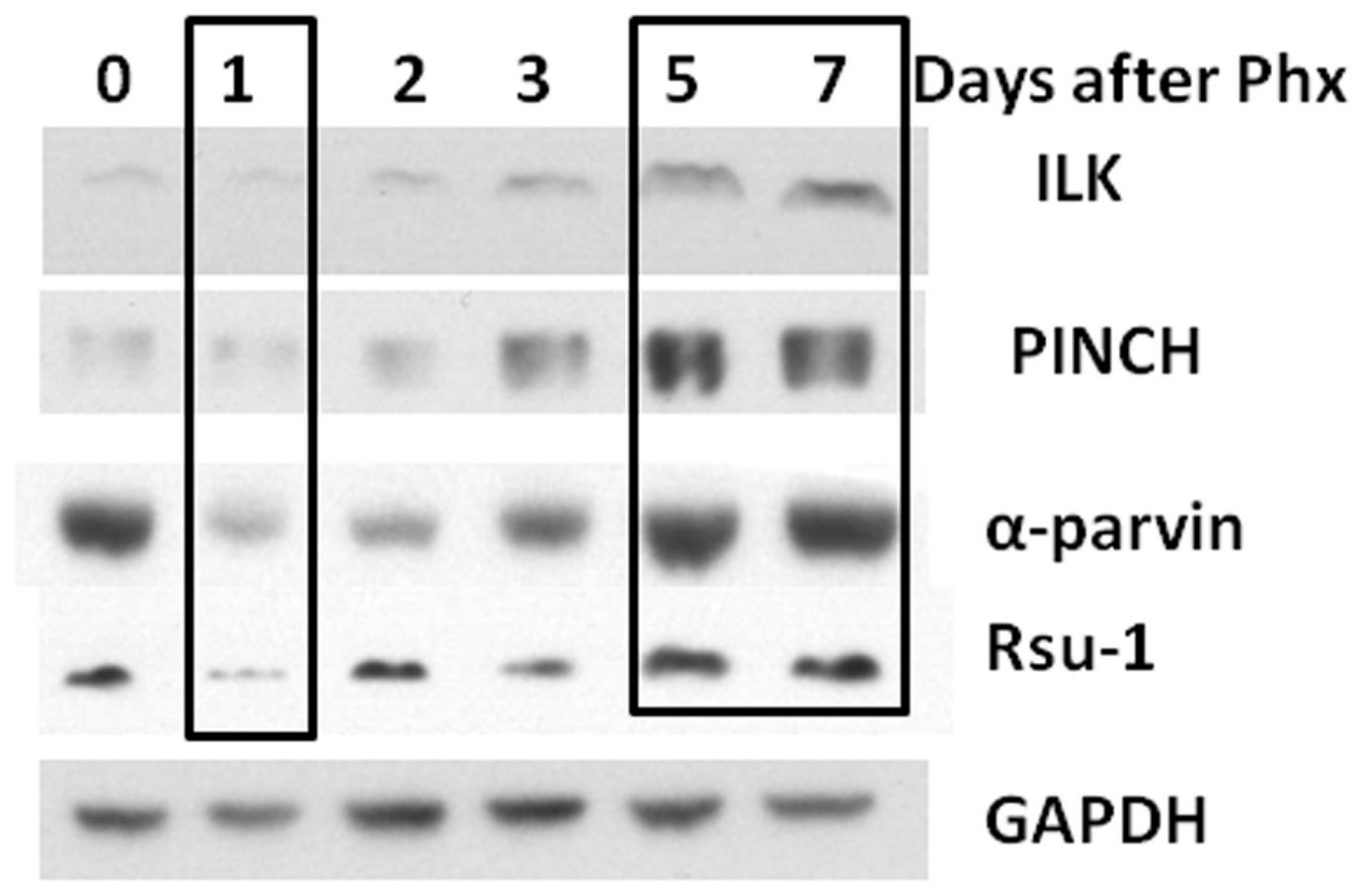
Components of the IPP complex are upregulated at the end of liver regeneration. Western blot of various components of IPP complex in rat hepatocyte pellet at various time points after partial hepatectomy. Hepatocytes were isolated by 2 step collagenase perfusion.

We determined the protein expression of individual components of the IPP complex in the hepatocytes isolated by 2-step collagenase perfusion at different time points after partial hepatectomy (PH) in rats. All components of the IPP complex were downregulated at day 1 after PH when there is a peak in rat hepatocyte proliferation (Figure 1). There was an upregulation of expression of all components of IPP complex and Rsu1 at days 5 and 7 when regeneration proceeds towards ending [15]. These data show that all components of the IPP complex are upregulated during the termination phase of liver regeneration.

### PINCH removal from hepatocytes leads to downregulation of all components of the IPP complex

**Figure 2:**
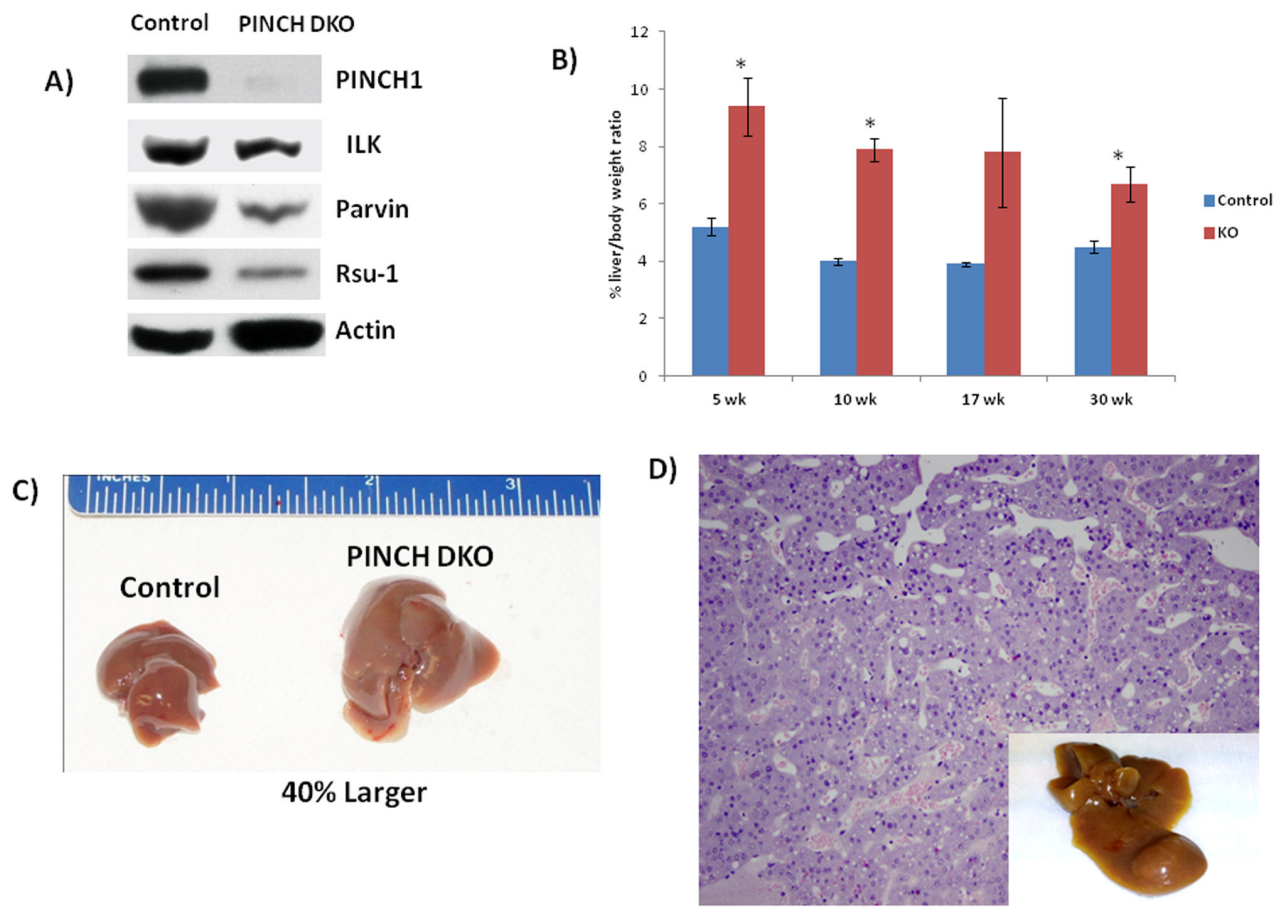
Morphological changes in PINCH DKO mice. A) Hepatocytes isolated from PINCH DKO mice show absence of PINCH1 (Note: PINCH2 is already systemically removed). Other components of the IPP complex were also downregulated. Hepatocytes were isolated from 17 week old mice. Western blot shows pooled samples from 3 mice. B) Percent liver weight to body weight ratios of control and PINCH DKO mice at different weeks of age. Each data point is the mean ± SE from more than three measurements per point. Data is expressed as means ± S.E. Comparison between two groups at the same time point is made by unpaired Student’s t test using Microsoft Excel. The criterion for statistical significance is p ≤ 0.05. * indicates statistically significant difference. C) Representative livers of control and PINCH DKO mice at 30 weeks of age indicating the difference in liver size between the two groups. D) Histology of the liver section of the PINCH DKO showing HCC. Representative livers of control and PINCH DKO mice at 1 year of age showing presence of liver tumors.

We first determined whether the PINCH1 gene was indeed deleted in the Cre expressing mice in which PINCH2 was deleted in germline. We carried out western blot analysis with anti-PINCH1 antibody in hepatocytes isolated from 17-week-old animals. We perfused livers with collagenase to isolate specific hepatic cell subpopulations and subsequently separated the hepatocytes from the nonparenchymal cells. As shown in Figure 2A, PINCH1 was knocked down efficiently in PINCH DKO mice. We next determined whether removal of PINCH from hepatocytes had any effect on the IPP complex. Levels of ILK were marginally decreased in the PINCH DKO mice while the level of Parvin was markedly decreased (Figure 2A). Rsu-1 which is known to bind to PINCH1 of the IPP complex [11] was also markedly decreased in the PINCH DKO mice (Figure 2A). These studies show that removal of PINCH from the IPP complex results in decreased expression of all components of the IPP complex.

### PINCH removal from hepatocytes results in histological abnormalities, sustained proliferation of hepatocytes, increased liver size and development of spontaneous tumors

We sacrificed mice at different ages after birth starting from 5wk, 10 wk, 17wk, 30 wk and 1 year. We examined the livers both morphologically and histologically. The liver to body weight ratio of the PINCH DKO mice was always higher than the controls at all ages (Figure 2B, Table 1). Final liver weight by the end of 30 weeks is 40% larger than the Control mice (Figure 2C). These mice were then followed till one year to see if the PINCH DKO developed spontaneous tumors. By 1 year 30% of the PINCH DKO mice developed spontaneous tumors (n=10) (Figure 2D). We did not observe any tumors in the control mice. The observed tumors were well differentiated hepatocellular carcinomas (Figure 2D).

**Table 1:** Individual liver and body weights of PINCH DKO and control mice.

**ControlMice**												
**Mice ID**	**Age**		**Body Weight**		**Liver Weight**		**% Ratio**		**Average**		**SD**	**SE**
c4	5 wk		27.7		1.6		5.78		5.26		0.57	0.33
c5	5 wk		28		1.5		5.36					
c6	5 wk		28		1.3		4.64					
c7	10 wk		31.5		1.2		3.81		4.02		0.23	0.11
c8	10 wk		33		1.4		4.24					
c9	10 wk		31		1.3		4.19					
c10	10 wk		28.6		1.1		3.85					
c1	17 wk		39.2		1.6		4.08		3.90		0.16	0.09
c2	17 wk		34		1.3		3.82					
c3	17 wk		31.6		1.2		3.80					
c11	30 wk		34.7		1.7		4.90		4.74		0.37	0.21
c12	30 wk		44		1.9		4.32					
c13	30 wk		44		2.2		5.00					
**PINCHDKOmice**												
**Mice ID**		**Age**		**Body Weight**		**Liver Weight**		**% Ratio**		**Average**	**SD**	**SE**
p15		5wk		26.5		2.5		9.43		9.43	1.86	1.07
p16		5wk		19.5		2.2		11.28				
p17		5wk		18.5		1.4		7.57				
p7		10 wk		31		2.2		7.10		7.92	0.85	0.43
p8		10 wk		33		3		9.09				
p9		10 wk		34		2.7		7.94				
p10		10 wk		34.4		2.6		7.56				
p1		17 wk		37.5		2		5.33		7.81	3.3857888	1.9548434
p2		17 wk		35.8		2.3		6.42				
p3		17 wk		36		4.2		11.67				
p11		30 wk		39		3.2		8.21		6.96	1.24	0.62
p12		30 wk		40		2.8		7.00				
p13		30 wk		38		2		5.26				
p14		30 wk		38		2.8		7.37				

**Figure 3:**
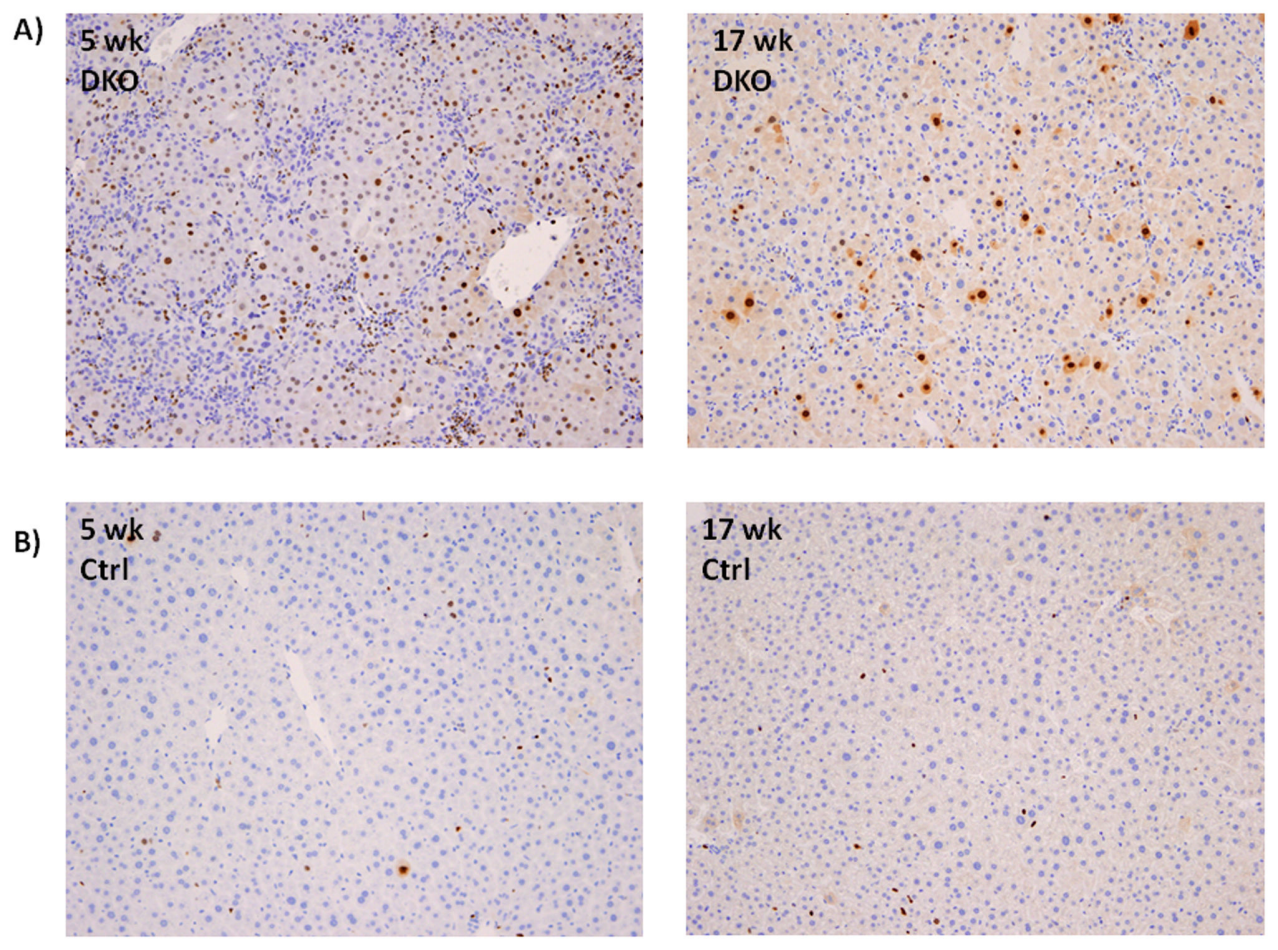
Increased hepatocytes proliferation in PINCH DKO mice. A) Ki67 positive hepatocytes in a 5 and 17 wk old PINCH DKO respectively. B) Ki67 positive hepatocytes in a 5 and 17 wk old control mice respectively.

**Figure 4:**
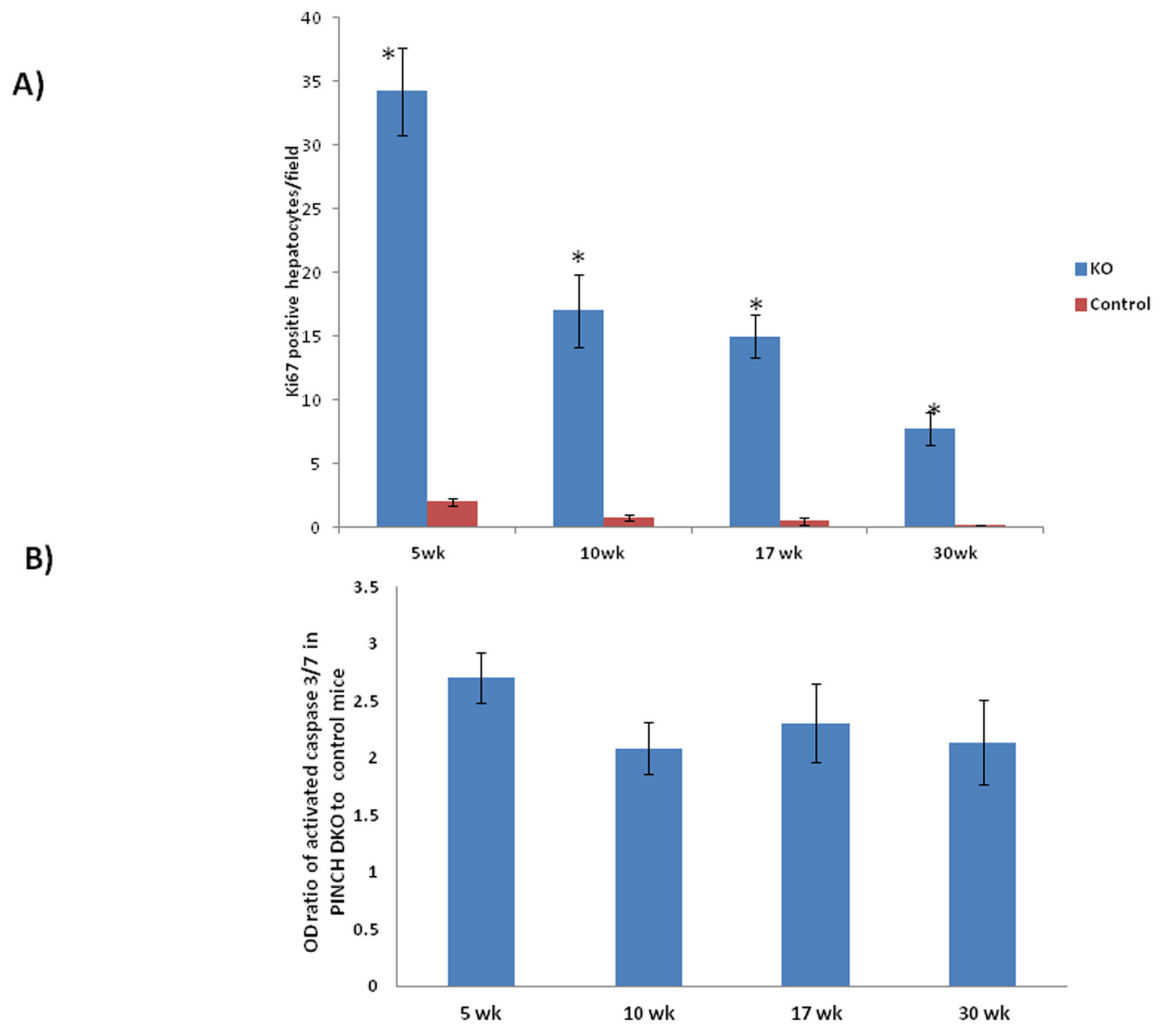
Quantitative assessment of hepatocyte proliferation and apoptosis in PINCH DKO mice. A) Number of Ki67 positive cells/field at different ages after birth. Each data point is the mean ± SE from two fields per slide from each animal in a total of at least 3 animals. B) Fold change in apoptosis (caspase3/7 activity) in the PINCH DKO cell lysates as compared to the controls at different ages. The numbers were derived as the ratio of caspace 3/7optical density between DKO and control mice. Each data point is the mean ± SE of at least 3 pairs of mice per time point. Comparison between two groups at the same time point is made by unpaired Student’s t test. The criterion for statistical significance is p ≤ 0.05. * indicates statistically significant difference.

**Figure 5:**
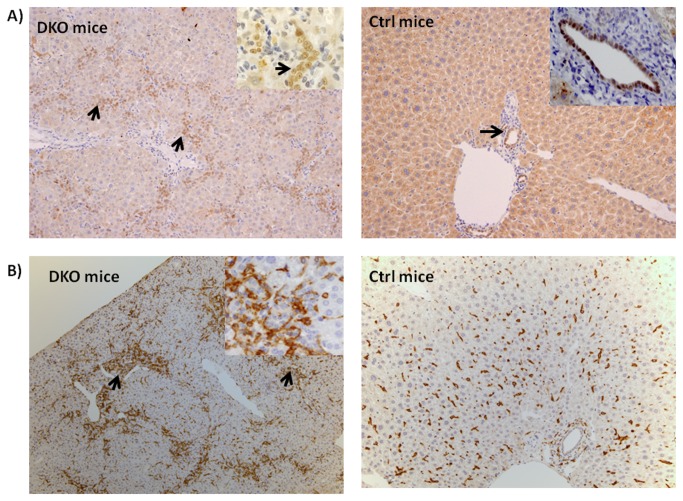
Histological Changes in PINCH DKO mice. A) Proliferation of biliary ductules was observed in a 17 wk PINCH DKO (DKO mice) and control mice (Ctrl mice). Expression of the biliary specific transcription factor HNF1β is shown in the nuclei of portal ductules (thick black arrow) as well as in the biliary cells proliferating and forming extra portal tracts (thick black arrows). Insert shows higher magnification (200X) ofHNF1β positive cells. Control mice show HNF1β positivity only in the bile duct (arrow). The staining seen in the control mice in the parenchyma is background stain. B) Increased inflammation in PINCH DKO mice. A 17 week old PINCH DKO mice showing increased F4/80 positive macrophages (shown by arrows) as compared to control mice. All the figures are 100X. The insert are of 200X magnification.

**Figure 6:**
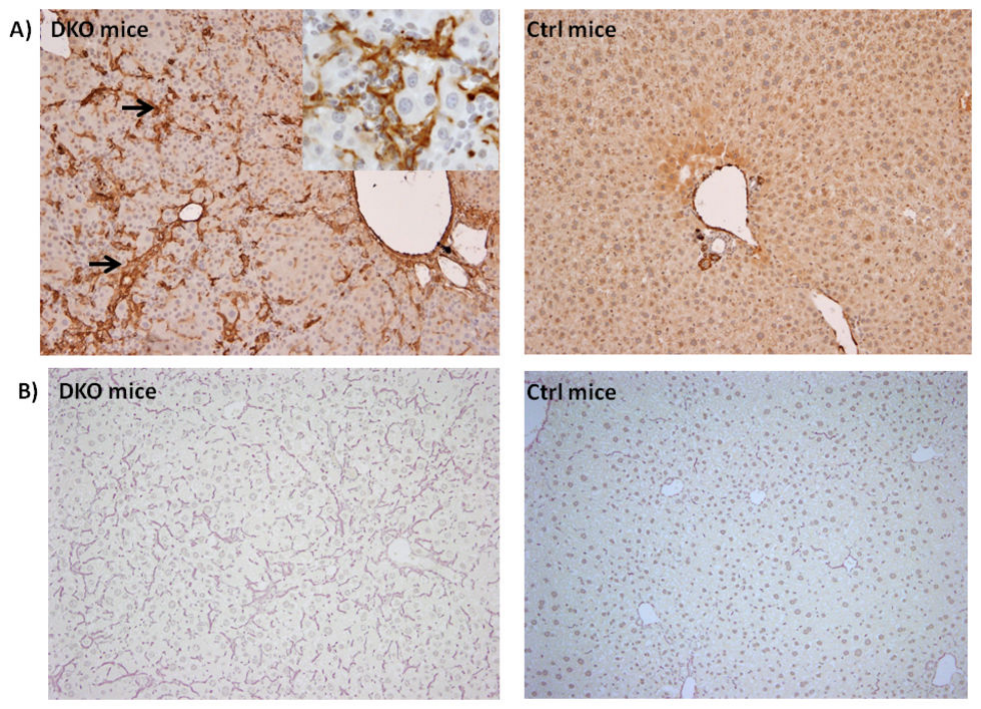
ECM changes in PINCH DKO mice. A) Photomicrograph of a liver of 5 week old PINCH DKO mouse showing increased stellate cell activation as evident by αSMA stain (shown by arrows). Similar photomicrograph from a Control mouse of the same age shows minimal activation of stellate cells B) Photomicrograph of a liver of a 30 week PINCH DKO mouse showing increased ECM deposition as evident by reticulin stain. All the figures are 100X. The insert are of 200X magnification.

**Figure 7:**
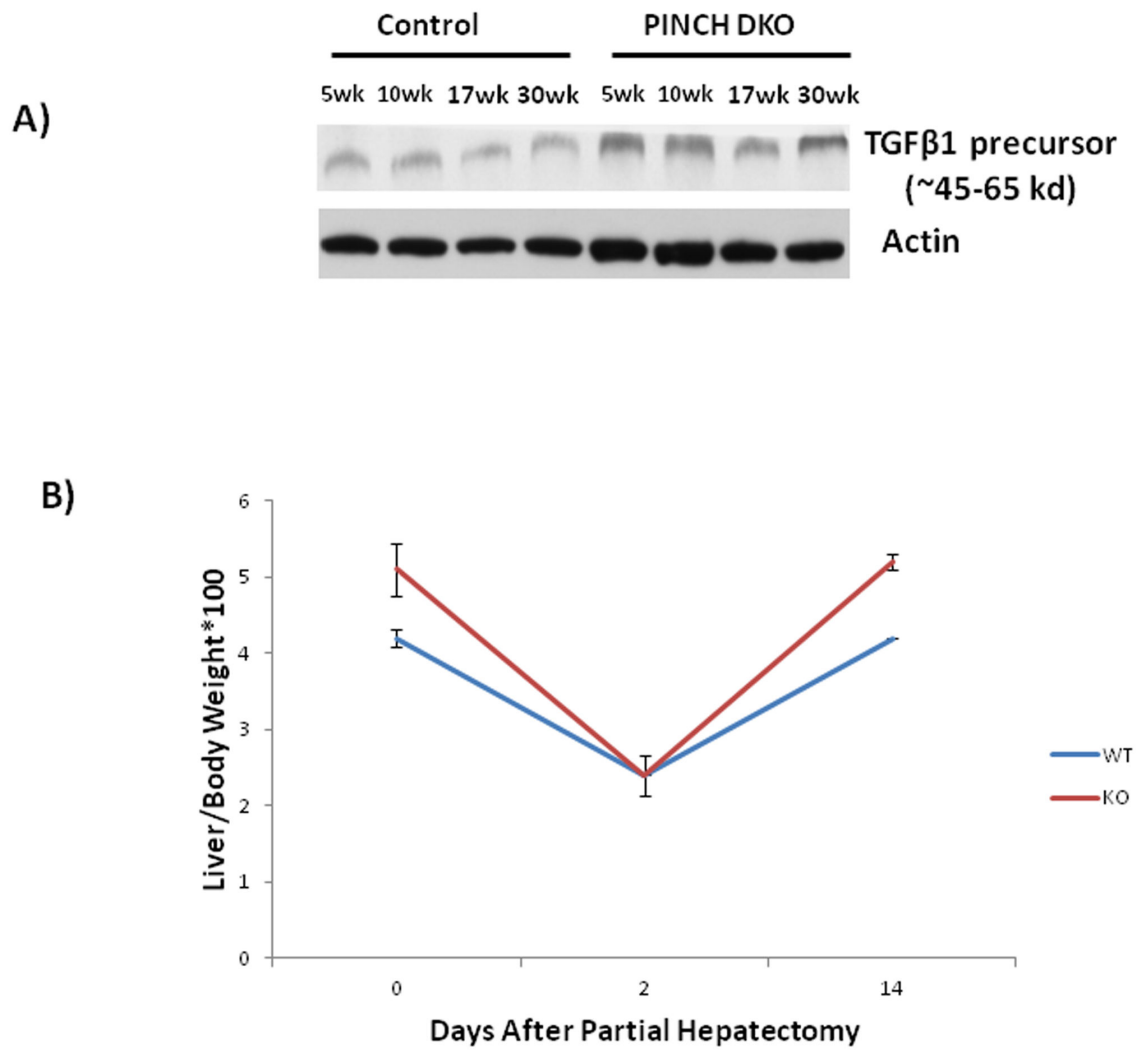
Liver regeneration kinetics in PINCH DKO mice. C) PINCH DKO mice show no termination defect. At Day 14 after partial hepatectomy, the percentage body/liver weight of the control and PINCH DKO mice reaches 100% of the original.

We monitored the cell kinetics in Control and PINCH DKO mice at 5, 17, and 30 weeks of age. The data in Figures 3 and 4 show that PINCH DKO mice had higher percent of hepatocytes in the cell cycle (Ki67-positive nuclei); and apoptosis (as assessed by caspase 3/7 activation) as a function of age of the mice. In control livers, the percent of Ki67-positive hepatocytes declined with age. In livers with targeted elimination of PINCH, the percent of Ki67-positive cells remained elevated with eventual decline by 30 weeks (Figures 3 and 4A). The apoptosis of hepatocytes (as assessed by caspase 3/7) remained almost 2-fold higher in the DKO mice at all times than in control livers (Figure 4B). In addition, extensive proliferation of biliary epithelial cells was observed in PINCH DKO mice, (Figure 5A). These cells were characterized as biliary by virtue of their morphology and the fact that their nuclei were staining positive for the transcription factor HNF*β*1 (Figure 5A). Proliferation of biliary cells started at 5 weeks, culminated at 17 weeks, and subsided by 30 weeks. The number of macrophages (as evidenced by F4/80 antibody staining) was also elevated between 5-17 wk but subsided by 30 weeks (Figure 5B). The tracts containing biliary epithelial cells also contained αSMA positive stellate cells (Figure 6A) suggesting increased activation of stellate cells in the PINCH DKO mice especially at 5 weeks of age (Figure 6A). There was progressive deposition of fine connective tissue material in the PINCH DKO livers (staining positive with silver stain for reticulin, Figure 6B). The livers of the PINCH DKO mice were also very hard and stiff due to increased ECM deposition. Deposition of excessive ECM was diffuse, surrounding individual hepatocytes (Figure 6B). There was no distortion of the portal to central axis of the lobule, nor any nodule formation etc, as seen in cirrhosis. There was an overall decrease in ECM deposition from 17 to 30 weeks. None of these changes were seen in livers of Control mice (PINCH2^-/-^ PINCH1 ^loxP/loxP^ Cre^-^). We also found increased expression of TGFβ1 precursor in the PINCH DKO mice (Figure 7A). We were not able to detect any activated TGFβ1 (twelve point five kd). We speculate that this might be due to its rapid utilization.

### PINCH DKO mice regenerate normally

We next studied liver regeneration after 70% partial hepatectomy in these mice. Studies with ILK KO have shown that these mice have a termination defect after partial hepatectomy [6]. Since the PINCH DKO mice also had a phenotype very similar to the ILK KO mice we expected a very similar response. To our surprise PINCH DKO mice showed no difference in the liver regeneration kinetics as compared to the control mice (Figure 7B). These studies suggest that PINCH might be playing a role in regulation the size of a quiescent liver but unlike ILK does not seem to be involved in regulation of the final liver size after a regenerative stimulus.

### Role of Rsu-1-PINCH in regulation of hepatocyte proliferation

**Figure 8:**
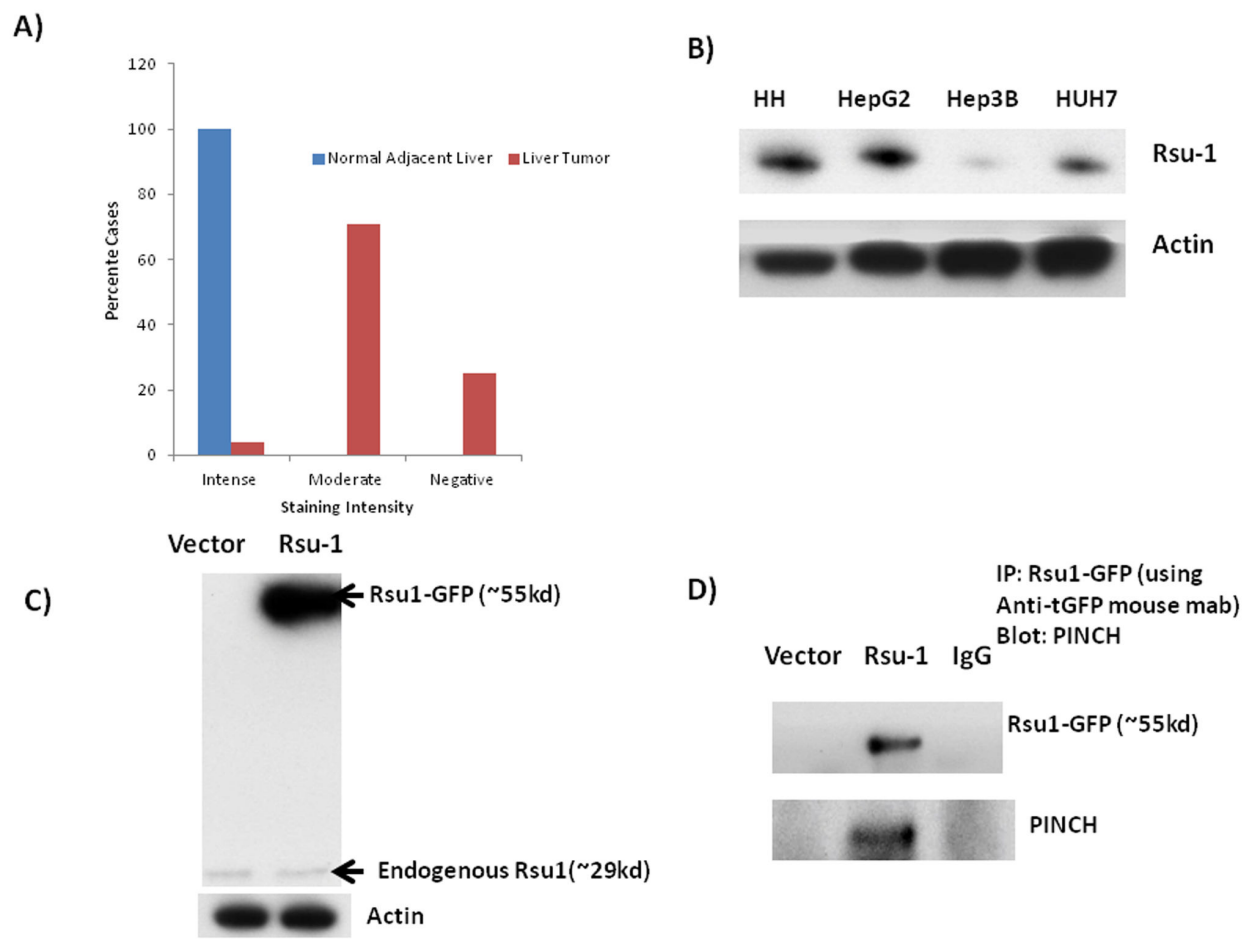
Rsu-1 levels in HCC. A) Graphical representation of the number of HCC cases positive, negative or moderately positive for Rsu-1 in HCC tissue array (24 cases/48 cores). B) Protein levels of Rsu-1 in HCC cell lines compared to human hepatocytes (HH). Most of the HCC cell lines show decrease in Rsu-1 protein. C) Successful overexpression of Rsu-GFP (fusion protein) in Hep3B cell line. GFP tagged ORF clone of Homo sapiens Rsu-1 (#RG203334, Origene) was transfected into Hep3B cell line and analyzed for Rsu-1 48 h after transfection. Since it is a GFP fused protein, the MW of Rsu-1 is ~fifty-five kd instead of twenty-nine kd (MW of GFP is ~twenty-six kd). D) GFP-Rsu-1 fusion protein associates with PINCH inside the cell. Overexpression of GFP-Rsu-1 in Hep3B cell line leads to association of GFP-Rsu-1 with PINCH. GFP was immunoprecipitated 48 h after transfection. GFP precipitates were probed with either GFP or PINCH. Presence of PINCH in GFP precipitates shows association of GFP-Rsu-1with PINCH.

**Figure 9:**
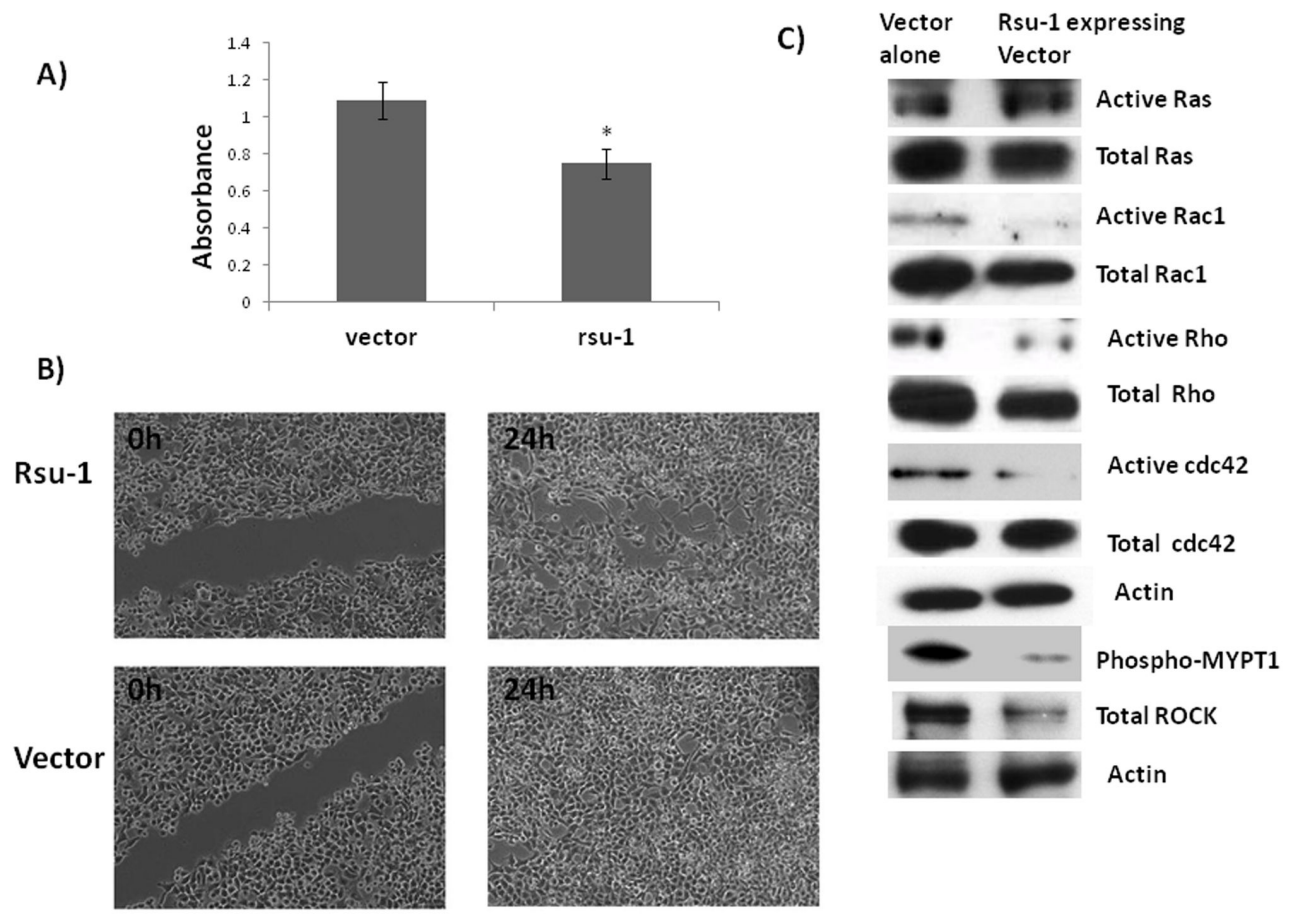
Functional significance of Rsu-1. A) Overexpression of Rsu-1 leads to a significant decrease in Hep3B proliferation. Hep3B cells were transfected with Rsu-GFP expression vector using Lipofectamine 2000 in OptiMEM. Twenty four hours after transfection, baseline MTT was measured in vector alone and Rsu-GFP expression vector. Proliferation was stimulated with 10% FBS in DMEM and MTT measured after 24 h. MTT values after 24 h were normalized to the controls. Data is expressed as means ± S.E from three separate experiments (two repeats). Comparison between two groups is made by unpaired Student’s t test. The criterion for statistical significance is p ≤ 0.05. B) Representative image from three separate experiments (two repeats) of wound healing assay in GFP-Rsu-1 overexpressing HUH7 cell line. Twenty four hours after cells were transfected with GFP-Rsu-1 expressing vector, a scratch was made with a sterile glass pipette and followed thereafter for 24 h. Cells were maintained in DMEM with 0.5% FBS. C) Rsu-1 overexpression inhibits Rho GTPase in Rsu-1 overexpression Hep3B stable transfectants. After transfection the cells were maintained on G418 (0.5mg/ml) for 7 days. At the end of 7 days cells lysates were prepared from 3 separate experiments and pooled for further analysis. GTP bound (active) RhoGTPases were measured by GST pull down assay. ROCK activity was measured by in vitro kinase assay by measuring the ability of ROCK to inactivate myosin phosphatase through the specific phosphorylation of myosin phosphatase target subunit 1 (MYPT1) at Thr696.

We next wanted to investigate the role of Rsu-1, the PINCH-selective binding partner, in the growth suppressive effects of PINCH and IPP complex. Rsu-1(Ras Suppressor-1) is a protein expressed in most cells [16] and has a leucine rich repeat (LRR) domain. It binds to PINCH1 of the IPP complex [11]. It is known to suppress Ras related functions in part through its association with the IPP (Integrin linked kinase-PINCH-Parvin) complex [11]. Studies have shown that Rsu-1 has suppressive effects on growth of cancer cells namely glioblastoma and mammary cells [11,12,17]. From a totally separate study in our lab utilizing liver cancers, we also found that 10% of the HCC patients had deletions in Rsu-1 gene [13] further strengthening our hypothesis that Rsu-1 might be a major negative growth regulator for hepatocytes. Rsu-1 levels were also lower in PINCH DKO mice (Figure 2A). Using tissue microarray (catalogue # LV481, Biomax US) we also found that most of the HCC cases were either negative or moderately positive for Rsu-1. Out of the 24 tumor tissue 6 tumor tissues (25%) were completely negative for Rsu-1 while 17 of the tumors were moderately positive for Rsu-1 and only 1 was strongly positive for Rsu-1 (Figure 8A). All the “normal” adjacent liver tissue showed strong staining for Rsu-1. In addition, we screened several hepatoma cell lines for the levels of Rsu-1. We found that Hep3B cells had very low protein levels of Rsu-1 (Figure 8B). We chose this cell line for evaluating the functional significance of Rsu-1. GFP tagged ORF clone of human Rsu-1 (#RG203334, Origene) was used for overexpressing Rsu-1 in Hep3B cell line and analyzed for Rsu-1 48 h after transfection. Rsu-1 was successfully overexpressed (Figure 8C) in this cell line. It was also demonstrated that expressed Rsu-1 is actually associated with PINCH (Figure 8D). Overexpression of Rsu-1 in Hep3B cells led to reduced cell proliferation as evident by MTT assay (Figure 9A). Since Huh7 cells have high migratory capacity, we overexpressed Rsu-1 in this cell line and measure their migratory capacity using the scratch assay. Overexpression of Rsu-1 led to reduced migratory capacity of Huh7 cells (Figure 9B). These studies suggest that PINCH may regulate hepatocyte proliferation/migration in part through Rsu-1. This was further strengthened by the observation that overexpression of eGFP-Rsu-1 in Hep3B cells leads to association of eGFP-Rsu-1 with PINCH (Figure 8D). We further investigated the mechanism by which Rsu-1 inhibits hepatocyte proliferation and migration. We found that Rsu-1 does not affect the activation of Ras (as its name suggests) but instead inhibits the activation of downstream RhoGTPase targets of Ras, namely RhoA, Rac1 and cdc42 (Figure 9C). We also measured the downstream target of RhoGTPase, Rho-associated kinase (ROCK) [18,19,20,21]. ROCK mediates Rho signaling and reorganizes actin cytoskeleton through phosphorylation of several substrates that contribute to cell proliferation and migration [18,19,20,21]. We measured ROCK protein by Western blot and activity by *in vitro* kinase by measuring the ability of ROCK to inactivate myosin phosphatase through the specific phosphorylation of myosin phosphatase target subunit 1 (MYPT1) at Thr696. As expected overexpression of Rsu-1 led to decreased ROCK activity (Figure 9C) and ROCK protein. There was also a marked decrease in the total ROCK protein suggesting that Rsu-1 can regulate the expression of ROCK.

## Discussion

Our previous studies have shown that ILK, a member of the IPP complex acts as a negative regulator of hepatocyte proliferation [4,6]. The objective of this study was to investigate the functional significance of PINCH (another member of the IPP complex, directly bound to ILK) in hepatocyte proliferation. Our studies suggest that removal of PINCH from hepatocytes led to destabilization of the whole IPP complex as evident from downregulation of the rest of the members of the IPP complex (Figure 2A). This demonstrates that PINCH in hepatocytes plays an important role in stabilizing the whole IPP complex. Removal of PINCH *in vivo* from hepatocytes led to an increase in the size of the liver by about 40%. Interestingly, the liver growth did shut down after 40% growth suggesting that the pathways involved in regulation of liver size are redundant and that the IPP complex is not the only one operating in this process. We speculate that focal adhesion Kinase (FAK) (or other non-identified pathways) might be compensating for the loss of IPP complex. Apart from the IPP complex, FAK are known to be a major mediator of integrin signaling [22,23]. The increase in the liver size was due to prolonged proliferation of hepatocytes and biliary cells between 5-30 wk of age, as well as due to increased deposition of extracellular matrix. Increased proliferation was also accompanied by some degree of hepatocyte apoptosis, which raises the question whether apoptosis is the driving force behind increased hepatocyte and biliary proliferation or whether it a response to increased proliferation. Because these possibilities are not mutually exclusive, it is possible, that both phenomena may be occurring at the same time. Since there is an overall increase in liver size, we believe that the predominant effect of removal of PINCH1/2 in hepatoblasts by genetic elimination during embryonic development is the enhanced proliferation of hepatocytes (and biliary epithelial cells), occurring because of the removal of the proliferation-inhibitory effects of ECM [24,25]. Note that by 30 weeks PINCH DKO livers are almost 40% larger than the control mice. This would be unlikely if hepatocyte apoptosis was the primary driving force for the histologic changes seen. We also noticed formation of tumors by one year of age. Hepatocellular carcinomas frequently appear in situations of prolonged, chronic, hepatocyte proliferation and alterations in ploidy, along with progressive appearance of loss of heterozygosity might play a role [26]. We do not consider loss of function of IPP complex as sufficient by itself to generate hepatic neoplasms. Generation of liver tumors is a stochastic process. It takes a combination of cooperative genomic or epigenetic alterations to form hepatic (or any other) neoplasm. The emergence of tumors in the livers of the PINCH DKO mice suggests that the absence of IPP complex can play a contributory role in this process if other suitable genomic alterations are also present [13].

These mice also showed increased number of macrophages as well as activated stellate cells as evident by increase in F4/80 and α-SMA positive cells respectively. The reasons for the deposition of enhanced extracellular matrix are not clear, either in PINCH DKO or in our previous work with ILK KO. Presumably the matrix is deposited by stellate cells, due to their increased activation. Biliary cell proliferation is known to induce a proinflammatory cytokine response [27,28] leading to an increased Kupffer cell accumulation in the liver. Since Kupffer cells and stellate cells are known to be a major source of TGFβ1 [29,30] increased TGFβ1 production by Kupffer cells and stellate cells may lead to activation of stellate cells and hence increased ECM production. Increased expression of the TGF-beta precursor is shown in Figure 7A. The increased production of ECM in a time dependent manner could also explain the decrease of cell proliferation from 5-30 weeks in PINCH DKO mice. We have shown previously that hepatocytes in HGM media lose their characteristic gene expression patterns and proliferate under the influence of HGF and/or EGF. Addition of artificial extracellular matrix to hepatocytes in culture (e.g. Matrigel, Type I collagen gels) restores full differentiation and inhibits hepatocyte proliferation [5,24,31].

We next tested the response of PINCH DKO mice to partial hepatectomy. Since the phenotype of the PINCH DKO mice is very similar to the ILK KO mice [4] we had hypothesized that PINCH DKO mice would also show a termination defect as the ILK KO mice [6]. Interestingly, liver regeneration response was not different as compared to the control animals.

Recently, we performed a study to assess genetic alterations (deletions or amplifications) [13] in 96 HCC samples. We found that in those samples 10% of the cases had deletions in Rsu-1, the PINCH1 binding partner. Previous studies had shown that transfection of an Rsu-1 expression vector in U251cells causing increased Rsu-1 expression suppressed growth and prevent tumorigenicity of this cell line in an athymic mouse model [32]. Similarly expression of Rsu-1 driven by an expression vector in breast cancer cell line suppressed its growth and migration [11,12]. Thus, it is likely that PINCH acts as a growth suppressor in many cell types through Rsu-1. Moreover, Rsu-1 levels in PINCH DKO mice are markedly decreased (Figure 2A). There is no literature regarding the function of Rsu-1 in normal liver. In view of recognized difficulties with transfection of normal hepatocytes, we overexpressed Rsu-1 in hepatoma cell lines Hep3B and Huh-7 and found that it led to decreased proliferation and migration respectively. These studies suggested that Rsu-1 is a growth suppressor. We further investigated its mechanism of action. The protein is perceived in the literature as direct suppressor of Ras activation [33]. Rsu-1 was isolated and identified for its ability to suppress Ras transformation [33]. Interestingly, our studies revealed that Rsu-1 does not directly affect activation of Ras but inhibits Rho GTPase, a downstream target for Ras [34,35,36]. Several studies have demonstrated that pathways "downstream" of Ras contribute to transformation of cells in response to activated Ras. Most interestingly, activation of Rho GTPases appears to be responsible for induction of tumorigenicity in epithelial cells [35,37]. Thus, Rsu-1 does not inhibit the activation of Ras but inhibits the activation of downstream target of Ras namely, RhoGTPase.

**Figure 10:**
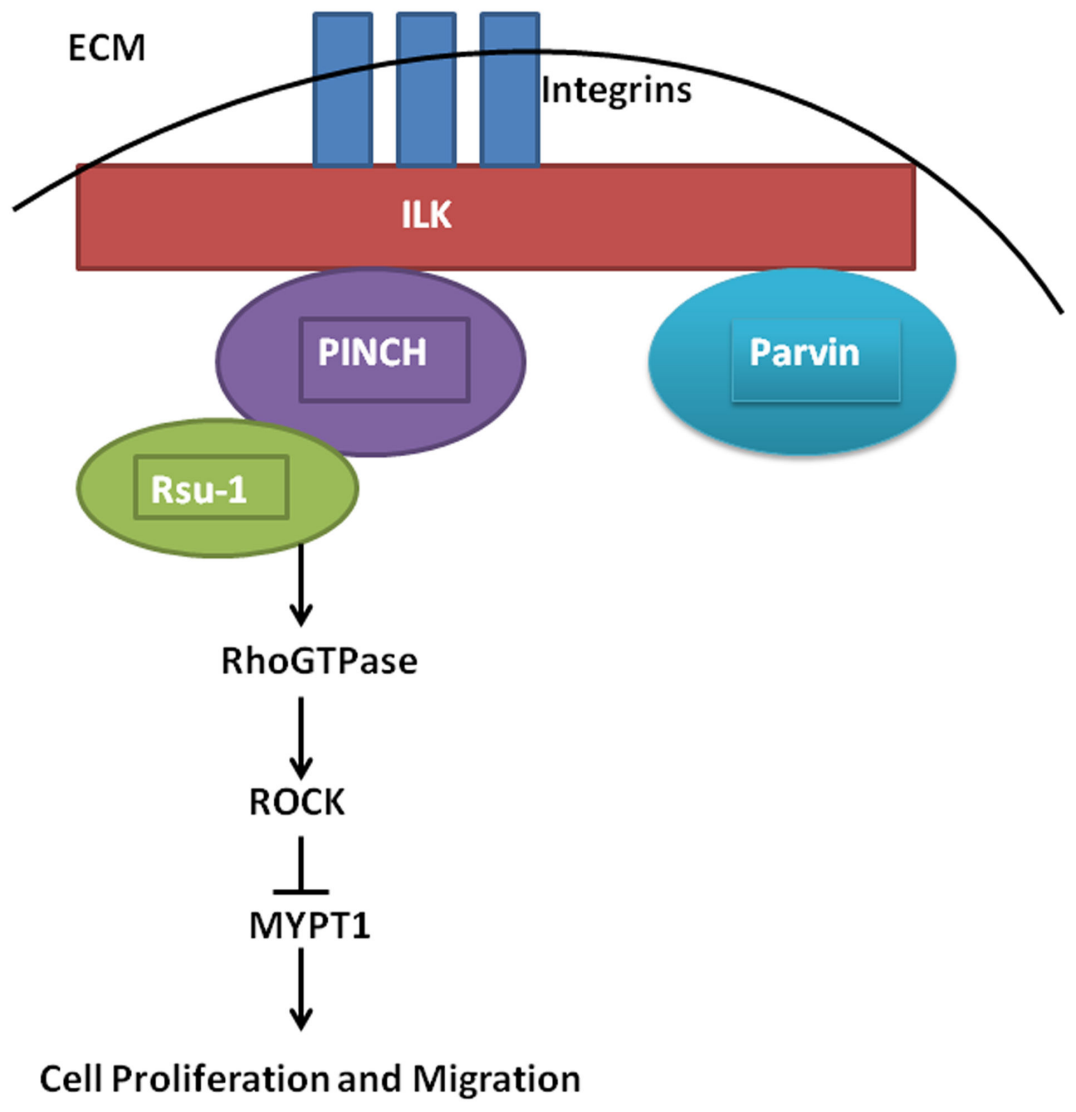
Model of how the Rsu-PINCH complex regulate hepatocyte proliferation through modulating RhoGTPase activity. We speculate that PINCH functions as a sensor to downregulate RhoGTPase signaling possibly by recruiting Rsu-1, a negative regulator of RhoGTPase. Suppression of RhoGTPase activity leads to decreased activation of the downstream target ROCK. ROCK regulates hepatocyte proliferation and migration by inactivating the myosin light chain phosphatase (MYPT).

We speculate that PINCH functions as a sensor to downregulate RhoGTPase signaling possibly by recruiting Rsu-1, a negative regulator of RhoGTPase. Removal of PINCH leads to diminished binding of Rsu-1 to PINCH and decrease in Rsu-1, as shown in our study. This leads to increased RhoGTPase activity leading to increased activation of the downstream target ROCK which in turn inactivates myosin phosphatase through the specific phosphorylation of myosin phosphatase target subunit 1 (MYPT1) at threonine residue 696 leading to increased cell proliferation and migration (Figure 10) [38]. The specific functions of the Rsu-PINCH complex need further investigation in the context of regulation of liver growth and carcinogenesis. Inhibition of Rsu-1 in cells with high background expression may be helpful to address its function further.

In conclusion, our studies suggest that removal of PINCH results in enlargement of liver and tumorigenesis. Rsu-1, a partner for PINCH and a protein often deleted in human liver cancer and markedly decreased in the PINCH DKO mice may play an important role in this process. It is also of interest that, given the growth suppressor effects of both ILK and PINCH (and Rsu-1), they all increase towards the end of regeneration, likely to contribute to the cessation of liver growth and the end of regeneration.
